# The Performance and Associated Risks of the Criteria for Sarcopenic Obesity Proposed by the European Association for the Study of Obesity in a Geriatric Population

**DOI:** 10.3390/nu16193315

**Published:** 2024-09-30

**Authors:** Begoña Molina-Baena, Alejandro Álvarez-Bustos, Jose Antonio Carnicero, Francisco José García-García, Leocadio Rodríguez-Mañas

**Affiliations:** 1 Department of Endocrinology and Nutrition, La Princesa University Hospital, 28006 Madrid, Spain; 2 Escuela de Doctorado, Universidad Autónoma de Madrid, 28049 Madrid, Spain; 3 CIBER of Frailty and Healthy Aging (CIBERFES), Institute of Health Carlos III, 28029 Madrid, Spain; a.alvarezbu@gmail.com (A.Á.-B.); lobouc3m@hotmail.com (J.A.C.); franjogarcia@telefonica.net (F.J.G.-G.); 4 Instituto de Investigación Biomédica La Paz (IdiPaz), 28029 Madrid, Spain; 5 Geriatric Research Group, Biomedical Research Foundation at Getafe University Hospital, 28905 Madrid, Spain; 6 Department of Geriatrics, Hospital Virgen del Valle, Complejo Hospitalario Universitario de Toledo, 45007 Toledo, Spain; 7 Department of Geriatrics, Getafe University Hospital, 28905 Madrid, Spain; 8 Servicio de Geriatría, Hospital Universitario de Getafe, Carretera de Toledo, Km 12.5, 28905 Getafe, Spain

**Keywords:** sarcopenic obesity, frailty, obesity, sarcopenia

## Abstract

Background: There is no gold standard definition of sarcopenic obesity (SO). Our objective is to evaluate the benefit of using the new definition proposed by the European Association for the Study of Obesity (EASO) in older people. Methods: Data from the Toledo Study of Healthy Aging, a study based on a cohort of community-dwelling older adults, were used. SO was defined according to the EASO and by a composite of the Foundation for the National Institute of Health (FNIH) for the diagnosis of sarcopenia and the WHO’s criteria for obesity (Body Mass Index, BMI ≥ 30 kg/m^2^; waist circumference, >88 cm for women and >102 cm for men). Frailty [Frailty Phenotype (FFP) and Frailty Trait Scale-5 (FTS5)] and disability (Katz Index) statuses were assessed at baseline and at the follow-up (median 2.99 years). Mortality at a 5-year follow-up was also assessed. The Logistic and Cox regression models were used to assess the associations. Results: Of the 1559 subjects (age 74.79 ± 5.76 years; 45.54% men), 30.15% (EASO/ESPEN) vs. 16.36% (FNIH) met the SO criteria (Kappa = 0.42). SO was associated with the prevalence of frailty by both the EASO’s [OR(95%CI): FFP: 1.70 (1.33–2.16); FTS-5 binary: 2.29 (1.60–3.27); β(95%CI): FTS-5 continuous 3.63 (3.00–4.27)] and FNIH+WHO’s criteria [OR (95%CI): 2.20 (1.61, 3.00)]. The FNIH + WHO’s criteria were cross-sectionally associated with disability [OR: 1.52 (1.07, 2.16); *p*-value 0.018], while the EASO’s criteria were not. The EASO’s criteria did not show any association at the follow-up, while the FNIH + WHO’s criteria were associated with incident frailty. Conclusions: The EASO’s new criteria for sarcopenic obesity demonstrate moderate agreement with the traditional definition and are cross-sectionally associated with adverse events, but they do not effectively predict the outcomes generally associated with sarcopenic obesity in older adults. Therefore, the performance of the EASO’s criteria in older people raises the need for refinement before recommending it for generalized use in this population.

## 1. Introduction

Sarcopenia has been defined as the presence of low muscle mass, strength, and/or performance [1]. Its prevalence varies between 10% and 27% in adults older than 60 [2], and its presence increases the risk of falls [3], frailty [4], disability, and mortality [5,6,7]. Several algorithms have been proposed in order to better capture this syndrome. Current criteria from organizations like the European Working Group on Sarcopenia in Older People (EWGSOP) emphasize low muscle strength as a primary criterion, supplemented by measures of muscle mass (e.g., via DXA). The addition of functional performance (e.g., gait speed) would identify individuals with severe sarcopenia [1]. On the other hand, the Foundation for the National Institutes of Health assess these same three criteria to evaluate it [8]. A global conceptual definition of sarcopenia valid for clinical and research settings was proposed by the Global Leadership Initiative in Sarcopenia (GLIS) [9]. According to this definition proposed in a Delphi survey, muscle mass, muscle strength, and muscle-specific strength are all accepted as ‘components of sarcopenia’, whereas impaired physical performance is accepted as an ‘outcome’ rather than a ‘component’ of sarcopenia [9]

Sarcopenic obesity (SO) is defined as the coexistence of excess adiposity and a reduction in muscle mass and function [1,2,3]. This condition increases the risk of adverse events, such as disability or mortality [4,5], and is of increasing clinical and research interest.

Although the prevalence of this syndrome was established at 11% among older adults [10], multiple factors such as age, being female, the presence of different pathologies, or socioeconomic level have been associated with a higher prevalence of SO [10,11,12,13,14]. In addition to this fact, there is a lack of a universal definition for SO, which hampers research and clinical practice, but recent efforts have been made to include measures of muscle strength, physical function, and fat distribution in the criteria [13,15,16]. The new diagnostic criteria, developed in collaboration by the European Association for the Study of Obesity (EASO) and the European Society for Clinical Nutrition and Metabolism (ESPEN) in 2022, represent a significant advancement in addressing this complex issue [17]. These criteria aim to facilitate the accurate identification of patients with sarcopenic obesity and establish a uniform set of diagnostic criteria through a common tool. Its diagnosis involves considering risk factors along with elevated values in Body Mass Index (BMI) or waist circumference, supported by clinical symptoms or evaluations through validated questionnaires [17]. However, no study to date has explored the association of this definition with adverse events in older adults, a population with a higher prevalence of SO, nor explored its superiority over previous definitions.

Thus, the aim of this study is to explore the predictive ability of this new definition of SO in community-dwelling older adults and compare it with the previous criteria proposed by the Foundations for the National Institutes of Health (FNIH) [8,18], in addition to the BMI cut-off point proposed by the World Health Organization (WHO), in community-dwelling older adults. Diagnostic accuracy, sensitivity, and specificity, as well as predictive values, are evaluated, and it is investigated whether the new criteria offer better predictive capacity compared to previous criteria in relation to frailty, disability, and mortality associated with sarcopenic obesity.

## 2. Materials and Methods

### 2.1. Population of Study

#### 2.1.1. Participants

Participant data were obtained from the Toledo Study of Healthy Aging (TSHA) [19]. The TSHA is a prospective cohort study designed to analyze determinants of frailty in individuals over 65 years old residing in the province of Toledo, Spain. Its general characteristics have been extensively described in previous works [19]. The TSHA was performed following the Declaration of Helsinki and was approved by the corresponding ethics committee [19].

#### 2.1.2. Informed Consent

All participants signed an informed consent form at the time of recruitment.

#### 2.1.3. Cohort Data Disposal

The TSHA is an open cohort which involves new participants in each wave in an effort to overcome the attrition effect. Moreover, the assessment carried out in each one of the waves was not exactly the same. For example, Dual-Energy X-ray Absorptiometry (DEXA) was carried out only in the people participating in the second wave. Thus, for the current analysis, we included subjects with available data at wave 2 (2009–2011) and wave 3 (2014–2017) in which body composition, frailty, and the other covariates were measured. Wave 3 was carried out 2.99 (range 2.0–5.4) median years after wave 2 and was used in the current study to assess the outcomes (frailty and disability). The third adverse outcome, death, was assessed through several sources of data, as described later on.

### 2.2. Study Design

#### 2.2.1. Sarcopenic Obesity

- SO was evaluated at baseline and defined according to the EASO and to a composite of the FNIH’s criteria for sarcopenia (standardized to our population [18]) in addition to the WHO’s criteria for obesity (BMI ≥ 30 kg/m^2^) and waist circumference (>88 cm for women; >102 cm for men) to build the so-called Former Sarcopenic Obesity Criteria (FSOC) (Table 1).
- Participants’ body heights and weights were evaluated through an analog medical scale and a stadiometer, respectively. The BMI was calculated according to standard procedures (body weight in kg/height^2^ in meters) [20]. Waist circumference (WC) was evaluated with inelastic belt-type tapes (in cm).
- Body composition was determined using Dual-Energy X-ray Absorptiometry (DEXA) (Hologic, Discovery QDR Series, Bedford, MA, USA). DEXA scans were analyzed using Physician’s Viewer software (Apex System Software, version 3.1.2: Bedford, MA, USA).
- Gait speed encompasses walk at a usual pace over 3 m. The quickest of two performances was analyzed.
- Isometric handgrip strength (IHS) was evaluated with a JAMAR dynamometer (Sammons Preston Rolyan, Bolingbrook, IL, USA) according to international procedures. The highest (in kg) of three attempts performed was chosen [21].

#### 2.2.2. Frailty Status

Frailty was evaluated at baseline and at follow-up and operationalized using Fried’s Frailty Phenotype (FFP) [22] and Frailty Trait Scale 5 (FTS5) [23].

- FFP [22] consists of the evaluation of five criteria: unintentional weight loss, low levels of physical activity, low walking speed, low his, and exhaustion. FFP cut-off points fitted to our population were described previously [19]. According to this definition, participants were classified as robust (no criteria), pre-frail (1–2 criteria), or frail (≥3 criteria). To assess frailty, both cross-sectionally and longitudinally, we pooled the pre-frail+frail categories due to the small number of frail individuals.
- FTS5 assesses frailty by evaluating BMI, 3 m gait speed, grip strength, self-reported physical activity, and balance [23] (Supplementary Table S2). Since FTS5 allows us to assess the frailty of individuals in a dichotomous (non-frail ≤ 25; frail > 25 points) and continuous manner, we assessed frailty according to this scale in both ways.

The occurrence of frailty in those who were categorized as robust (FFP) or non-frail (FTS5) at baseline was registered as incident frailty. In addition, an increase of 2.5 points or more independently of the FTS5 score at baseline was considered as worsening on this scale.

#### 2.2.3. Adverse Outcomes

In addition to frailty status, disability and mortality were considered.

##### Disability

- Disability was evaluated at baseline and at follow-up according to the Katz Index [24].
- Worsening disability was defined as the advent of a new disability in this tool.

##### Mortality

- Mortality was ascertained through hospital records, phone calls, and the National Death Index (Ministry of Health, Consumer Affairs and Social Welfare, Madrid, Spain). The follow-up time was set to 5 years.

#### 2.2.4. Covariates

- Comorbidity was evaluated according to the Charlson Comorbidity Index [25].
- Polypharmacy was defined as the use of >5 drugs.
- Educational level was recorded as non-educated, primary school incomplete, and primary school complete or superior.

### 2.3. Statistical Analysis

- Demographic and clinical characteristics were compared between groups defined by both sets of criteria. Descriptive variables were compared between SO statuses through Mann–Whitney U test or Chi-square test for continuous and categorical variables, respectively.
- Concordance between SO definitions was assessed using Kappa coefficients. Diagnostic validity measures such as sensitivity, specificity, and predictive values were calculated.
- Logistic regressions (frailty and disability) and Cox proportional hazard models (mortality) were performed to evaluate the association between SO, according to the definitions proposed by the EASO and FSOC, and negative health events. We further segmented our population into categories according to both SO definitions.
- For regression analysis, the following adjustment models were used: Model 1 (unadjusted), Model 2 (age and sex), Model 3 (age, sex, Charlson index, and polypharmacy), and Model 4 (age, sex, Charlson index, polypharmacy, and educational level).

Statistical analyses were performed using R version 3.6.1 for Windows (Cran R, Vienna, Austria). Statistical significance was set at a *p*-value < 0.05.

## 3. Results

One thousand five hundred and fifty-nine individuals were included (the mean age was 74.79 ± 5.76 yrs.; 45.54% male). The baseline clinical characteristics of the sample according to both sets of criteria for SO are presented in Table 2. A total of 470 (30.15%) and 255 (16.36%) subjects met the SO criteria proposed by the EASO and FNIH, respectively. Significant differences were found between SO status according to both definitions in terms of age, gender, body composition parameters, frailty, and disability.

### 3.1. Diagnostic Validity Analysis between SO Definitions

A comparison of the diagnostic performance metrics revealed a fair to moderate agreement between the EASO criteria and the FSOC (Kappa Index 0.42) (Table 3). Sensitivity and specificity were 75% and 79%, respectively, while the positive predictive value was 42%. The Youden index was 0.55, demonstrating moderate discriminatory capability for the EASO criteria over the FSOC (Table 3).

Because of this level of agreement, we decided to compare the four categories based on whether or not individuals met the two SO definitions. The characteristics of the individuals segmented on this basis are shown in Supplementary Table S2. A total of 197 (12.64%) individuals met the SO criteria according to both definitions, whereas 273 [17.51%, EASO (+)/FSOC (−)] and 58 [3.72%, EASO (−)/FSOC (+)] met the criteria on the basis of only one of the two. A total of 1031 subjects (66.13%) were not categorized as having SO regardless of the criteria used to define the SO condition. Significant differences between groups were observed in all comparisons performed.

### 3.2. Cross-Sectional Association between Sarcopenic Obesity, Frailty, and Disability

The presence of sarcopenic obesity regardless of the definition used significantly increased the risk of prevalent frailty independently of the scale used to assess frailty (Table 4). SO increased the risk of frailty according to FFP by 1.70 (EASO) and 2.20 (FSOC). As for FTS5, the risk increased by 2.29 (EASO) and 6.82 (FSOC) for frailty status and by 3.63 (EASO) and 6.57 (FSOC) for frailty score (*p*-value < 0.001 for all of the analyses).

However, when we evaluated the four categories according to sarcopenic obesity status, taking as reference individuals who did not meet the SO criteria according to either of the proposed algorithms, those who were identified by both definitions had a 2.79 (FFP) and 6.65 (FTS5) risk of being frail in addition to a higher score according to FTS5 [β(95%CI): 7.30 (6.41, 8.18); *p*-value < 0.001]. However, subjects who were categorized as having SO according to the new definition (EASO) only had a higher score on FTS5 [β(95%CI): 1.86 (1.12, 2.60); *p*-value < 0.001], but they did not present a higher risk of being frail by either definition in the fully adjusted model. On the other hand, older adults with sarcopenic obesity who were only identified by the FSOC definition had a higher risk of being frail and a higher score according to FTS5 (*p*-value < 0.001), but not for FFP (*p*-value 0.71).

Regarding disability status, both definitions of SO increased the risk of being disabled in the raw model. However, when covariates were added, only the FSOC definition increased the risk of this condition [OR (95%CI): 1.52 (1.07, 2.16); *p*-value 0.018]. As for comparisons between categories, only those identified by both definitions increased the risk of being disabled [OR (95%CI): 1.55 (1.05, 2.28); *p*-value 0.026]. The EASO (+)/FSOC (−) category was not related with disability in any of the proposed models, while EASO (−)/FSOC (+) was only associated with this condition in the bivariate model.

### 3.3. Longitudinal Associations between Sarcopenic Obesity and Adverse Events

At the follow-up assessment, 44 (FFP) and 94 (FTS5) individuals developed frailty. In addition, 457 worsened by at least 2.5 points on FTS5, while 315 worsened in disability. There were 137 deaths. Significant differences were found with respect to the rates of incident frailty and worsening frailty according to the FSOC definition of sarcopenic obesity but not according to the EASO’s definition (Supplementary Table S3). With respect to the categories according to both definitions, differences were found only in incident frailty (Supplementary Table S4).

### 3.4. Frailty

FSOC significantly increased the risk of incident frailty [Model 4; FFP, OR (95%CI): 1.70 (1.05, 2.75), *p*-value 0, 031; FTS5, OR (95%CI): 2.94 (1.74, 4.94), *p*-value < 0.001] and worsening in frailty by FTS-5 [OR (95%CI): 1.67 (1.12, 2.47); *p*-value 0.011] (Table 5). On the other hand, the new definition proposed by the EASO was only associated with incident frailty by FFP in the bivariate model, but not in any adjusted analyses.

Based on categories, EASO+/FSOC+ showed a significant association in Models 1, 2, and 3 for incident frailty according to FFP and reached close to significance in Model 4 (*p*-value 0.058). This association was significant for incident frailty for FTS5 [OR (95%CI): 2.39 (1.30, 4.40); p-value 0.005], but not for worsening frailty (*p*-value 0.11). EASO+/FSOC− was not associated with incident frailty in any of the models evaluated. On the other hand, EASO−/FSOC+ was associated with incident frailty and worsening for FTS-5.

### 3.5. Worsening Disability and Mortality

Regarding worsening disability, the definition of FSOC increased the risk by 43% in the bivariate model (*p*-value 0.031). However, this association became non-significant in the adjusted models. This was also the case for the EASO (−)/FSOC (+) category, which was only associated with worsening disability in the crude model. Neither the definition proposed by the EASO nor the rest of the categories was associated with disability in any of the models studied.

Regarding 5-year mortality, neither definition had a significant association with the event. However, in terms of categories, only those who were identified by the FSOC, but not by the EASO criteria, increased their risk of death regardless of the covariates included in the model [HR(95%CI): 2.28 (1.05, 4.96); *p*-value 0.038] (Table 6).

## 4. Discussion

Our study presents an initial effort to evaluate and validate the criteria for diagnosing sarcopenic obesity in an older population by incorporating the proposal introduced by the EASO/ESPEN consensus expert group. To achieve this, we compare this definition with a previous one, combining the criteria proposed by the FNIH (sarcopenia) and the WHO (obesity) in a large cohort of Spanish community-dwelling older adults. Our findings reveal substantial heterogeneity among the definitions in terms of prevalence [30.15% (EASO/ESPEN) vs. 16.36% (FNIH)] and their association with adverse events. One of the potential explanations for the discrepancies in predicting adverse events and the low concordance between both definitions is the difference in how the two components of the condition (i.e., sarcopenia and obesity) are defined. According to our results, the FSOC are stronger predictors of frailty and disability, both cross-sectionally and longitudinally, than the new criteria.

Despite the possible clinical relevance that sarcopenic obesity could have in the assessment and management of older adults, the diverse criteria used to diagnose it have hindered its effective treatment as a health issue. Prior to the definition proposed by the EASO/ESPEN, the overall prevalence of SO was 11% (95% CI, 10–13%) using various definitions, cutoff points, techniques, and population settings [17]. In our population, 470 (30.15%) and 255 (16.36%) subjects met the criteria for SO according to the EASO’s and FNIH’s criteria, respectively, with a positive screening in nearly 90% of the tested individuals. The fact that 90% of the individuals met the screening criteria and had to be evaluated for diagnostic criteria, which identifies one in three adults over 65 years of age as having sarcopenic obesity, raises the question of whether this definition is valid for this population, especially considering that its association with negative health events, especially at the prospective level, seems at least superfluous and does not exceed the criteria proposed above. In addition, the FSOC presented a higher concordance with frailty despite the fact that the new EASO/ESPEN criteria include many variables very similar to those included in Fried’s phenotype, which raises the question of whether they really try to capture sarcopenic obesity or a mix of geriatric syndromes with no real ability to predict adverse events.

It is important to note that the definition proposed by the ESPEN-EASO is quite lax when determining the variables of sarcopenia, obesity, and sarcopenic obesity itself. In this sense, while sarcopenia includes muscle mass and strength, obesity focuses on excessive fat tissue, and sarcopenic obesity is defined as the coexistence of excess adiposity and reduced muscle mass/strength. Nevertheless, this definition does not provide specific cut-off points as the other definition does. To construct our ESPEN-EASO definition, we used the cut-off points provided in this manuscript which were proposed in a Caucasian population. Nevertheless, these cut-off points have not been validated in our population as the ones used to defined sarcopenia in the FSOC’s definition were [18]. On the other hand, the sarcopenia item in the EASO-ESPEN definition does not include gait speed, though this item is included in the FNIH’s definition. In this sense, the European Working Group on Sarcopenia (EWGSOP) used these criteria to identify ‘severe sarcopenia’, a fact which may be crucial in the differences between the two definitions.

To date, the criteria for defining sarcopenic obesity typically involve the appendicular muscle mass-to-body weight ratio and the fat mass-to-body weight ratio [26]. In our study, we used the criteria proposed for the FNIH Sarcopenia Project (National Institute on Aging), designed to address the growing concern about sarcopenia, an age-associated syndrome characterized by the presence of low muscle mass and function [8]. However, different definitions of sarcopenia may present similar associations with different outcomes [3,7,27], and some studies have found differences based on the definition used [28], increasing the need to standardize these criteria [1].

In addition to the sarcopenia definition, we used the WHO-supported criteria for defining obesity (BMI ≥ 30). It is well known that in older adults, BMI does not accurately represent anthropology. Nevertheless, its rapidity and ease of assessment allow it to continue to be evaluated in clinical practice. In this sense, this marker is also a matter of debate because different studies have found controversial information with different anthropometric markers [29,30,31]. In fact, some authors have proposed other cut-off points to identify obesity in this population [29,31,32]. In addition, this cutoff point could vary depending on the adverse event studied. For example, it is possible that the risk of disability starts to increase from a BMI of 28 [32], while the risk of death starts to increase from 33 [31,32]. In fact, a recent systematic review proposed that obesity impairs older adults’ physical function with sarcopenia but increases their survival prognosis [33]. For this reason, it may be necessary to supplement certain assessments, such as the waist circumference, to capture the negative effects of excess fat tissue in older adults. In this sense, ABSI (A Body Shape Index), a measure of body composition that relates waist index to BMI and height, has been proposed as a proxy for sarcopenic obesity [34,35]. Taking into account the shortcoming of BMI to define obesity appropriately in older people, we also used the criterion of abdominal perimeter. Both criteria have been shown to predict functional deterioration in older people [36]. In fact, the relationship between BMI and frailty could be influenced by abdominal adiposity assessed by WC [36].

In the current geriatric framework in which function prevails over disease [37], our results support the evaluation of sarcopenic obesity as a prelude to entities such as frailty and disability. However, few studies have evaluated the association between sarcopenic obesity and frailty. In fact, the current evidence is that there are hardly any studies that associate both entities in a cross-sectional manner [38,39,40]. It is true that sarcopenia, independently of obesity, seems to favor the metabolic environment that precedes frailty [4,41,42,43,44], while the interaction of both entities seems necessary for an individual to worsen in their 3-year disability [5]. Nevertheless, according to our results, sarcopenic obesity does not increase the risk of disability at 3 years. Given that in our population, sarcopenia has been associated with disability in this time period [5,7], it is possible that obesity does not add any risk for this event, at least according to our definition. Future articles should evaluate this aspect.

Furthermore, in our population, we found no significant association between sarcopenic obesity and 5-year mortality. In fact, we found that the category that included FNIH+/EASO− presented an increased risk of mortality versus our reference category. We performed a sensitivity analysis by increasing the follow-up time to 6 and 7 years and found that this association was lost once the confounding factors included in the study were included, so we conclude that this is a spurious result but that it could certainly be of interest if confirmed in other populations. In a systematic review published by Zhang and colleagues [45], they observed that the risk of death attributable to sarcopenic obesity in adults living in the community was HR (95%CI): 1.14 (1.06–1.23). Nevertheless, in 8 of the 14 articles included in this sub-analysis, the presence of OS did not increase the risk of death. This fact could be justified not only by the definition chosen to evaluate obesity, but also by the follow-up time employed. In our study, we decided to set the follow-up time to 5 years. Future studies should explore whether this association could differ with different follow-up times.

The strengths of our study are that the TSHA is a well-categorized cohort of older adults in which the assessments were performed by experienced health professionals. In addition, we included two frailty scales, previously standardized to our population, as well as the definition of sarcopenia. Likewise, body composition was assessed by DEXA, which is considered the gold standard. On the other hand, we used a cross-sectional and longitudinal design with frailty and disability as well as the exhaustive verification of mortality within the cohort. Finally, the categorization of the population into four categories according to the criteria used in the literature and the current ones proposed by the EASO/ESPEN allowed us to increase the sensitivity of our observations. Regarding the limitations, it is important to note the lower prevalence of frailty according to the FP than in the entire TSHA due to some individuals not being able to attend the DEXA evaluation. Additionally, some categories formed on the basis of both definitions of sarcopenic obesity present a moderate number of individuals. In fact, the FNH+/EASO− category only includes three male individuals, which could interfere with the results.

On the other hand, it is important to note that these conclusions are based on the older population studied in this comparative analysis. Given that sarcopenic obesity can affect individuals of all ages, it is possible that the new criteria may be useful in other populations (i.e., inpatients, older adults, or cancer population) as well. However, further research is necessary to establish the diagnostic validity of these criteria in routine clinical practice across different age groups.

The findings of our study have important clinical implications for the diagnosis and treatment of sarcopenic obesity (SO) in older adults, particularly concerning the use of the EASO/ESPEN’s criteria. Although these new criteria identify a larger proportion of individuals with SO, their ability to predict adverse outcomes over time is limited. Since frailty and functional impairment are critical concerns in geriatric care, future refinements to the EASO/ESPEN’s criteria should aim to improve their predictive power for these conditions. Specifically, incorporating functional measures, such as gait speed and grip strength, into the diagnostic framework could enhance their usefulness in clinical practice.

Furthermore, with the growing importance of the early detection of sarcopenic obesity to prevent disability and frailty, the criteria should emphasize body composition assessments beyond BMI. Measures such as the waist-to-hip ratio or advanced imaging techniques like DXA (Dual-Energy X-ray Absorptiometry) or BIA (Bioelectrical Impedance Analysis) offer a more detailed view of fat distribution. These adjustments could increase the sensitivity of the criteria to the specific needs of geriatric populations and improve their effectiveness in screening individuals at high risk of frailty. Additionally, incorporating markers of inflammation or metabolic dysfunction may help capture the full extent of the disease and its impact on health. This is also the case for bone. Some studies have already proposed that bone, in addition to obesity and sarcopenia, could increase the risk of certain adverse events [46]. This is the case, for example, for frailty, where some studies have already evaluated the role of osteosarcopenic obesity at the cross-sectional level [47,48]. However, future studies should evaluate the role of this condition at the longitudinal level.

In summary, while the EASO/ESPEN criteria represent progress in defining SO, their application in clinical settings would benefit from further refinement to improve their reliability in predicting adverse health outcomes and guiding interventions aimed at preventing disability in older adults.

## 5. Conclusions

In summary, the new criteria proposed by the EASO for sarcopenic obesity identify a higher proportion of individuals with sarcopenic obesity than the FNIH’s criteria, presenting a low agreement between both definitions. Nevertheless, this new definition is associated with fewer cross-sectional outcomes than the former definition and does not predict incident negative health events in older adults. According to our results, the EASO’s criteria for defining SO should be reconsidered when identifying this syndrome in adults over 65 years of age. Further research is needed to understand the clinical implications of these differences and their impact on patient care.

## Figures and Tables

**Table 1:** Comparative key differences between ESPEN-EASO criteria and FSOC.

	EASO	FSOC
**Screening**	Screening: meet any of the following criteria	NA
	Age > 70 yrs	
	Obesity criteria: - WC > 88 cm women, >102 cm men - BMI ≥ 30 kg/m^2^	
	Depression	
	Positive CCV	
	eGFR < 60 mL/min/1.73 m^2^	
	STROKE/TIA	
	COPD/asthma	
	Charlson > 0	
	Meet any of the above criteria	
	Weakness, exhaustion	
	Fatigue	
	Perceived progressive limitations of movement	
	Acute illness/recent nutritional events	
	Recent hospitalization	
	Recent major surgery or recent trauma with/without complications	
	Recent recent sustained immobilization or reduced mobility	
	Recent history of reduced food intake	
	Unintentional weight loss (frailty phenotype criteria).	
**Diagnosis**		
**Low muscle mass**	ALM/weight: - M, <25.7% - W, <19.4%	ALM/BMI - M, <0.65 - W, <0.54
**Low grip strength**	Low handgrip strength (in kg) according to sex and quartile of BMI: - M, BMI_Q1: ≤19 - M, BMI_Q2: ≤22 - M, BMI_Q3: ≤22 - M, BMI_Q4: ≤22 - W, BMI_Q1:≤10.4 - W, BMI_Q2: ≤12 - W, BMI_Q3: ≤11 - W, BMI_Q4: ≤12	- M, <25.51 - W, <19.19
**Low muscle function**	NA	Gait speed < 0.8 m/s
**Presence of obesity**	- M, >27% fat mass - W, >38% fat mass	BMI: ≥30 kg/m^2^ WC: >88 cm women, >102 cm men

**ALM**: Appendicular Lean Mass; **BMI:** Body Mass Index; **CCV:** Cardiovascular Condition; **COPD:** Chronic Obstructive Pulmonary Disease; **eGFR**: Estimated Glomerular Filtration Rate; **FSOC:** Former Sarcopenic Obesity Criteria; **NA:** Not Applicable; **TIA:** Transient Ischemic Attack; **WC:** Waist Circumference.

**Table 2:** Demographic characteristics of study population according to EASO/ESPEN criteria and FSOC.

		EASO/ESPEN			FSOC		
**Variable**	**All**	**Sarcopenic Obesity**	**Non-Sarcopenic Obesity**	***p*** **-Value**	**Sarcopenic Obesity**	**Non-Sarcopenic Obesity**	***p*** **-Value**
**N**	1559	470	1089		255	1304	
**Age (years), mean (SD)**	74.79 (5.76)	75.20 (4.99)	74.61 (6.06)	**0.003**	76.86 (5.14)	74.38 (5.79)	**<0.001**
**Gender (male), n (%)**	710 (45.54)	179 (38.09)	531 (48.76)	**0.004**	36 (14.12)	674 (51.69)	**<0.001**
**Charlson index score, mean (SD)**	1.19 (1.63)	1.41 (1.70)	1.10 (1.59)	**<0.001**	1.45 (1.83)	1.14 (1.58)	**0.005**
**Number of drugs, mean (SD)**	4.89 (2.91)	5.81 (2.82)	4.49 (2.86)	**<0.001**	6.29 (2.92)	4.62 (2.83)	**<0.001**
**BMI, mean (SD)**	29.18 (4.56)	32.05 (3.74)	27.94 (4.32)	**<0.001**	33.05 (4.23)	28.42 (4.23)	**<0.001**
**DEXA parameters**							
**Total lean mass male (KG), mean (SD)**	50.74 (6.70)	53.48 (6.23)	49.81 (6.60)	**<0.001**	48.50 (5.20)	50.86 (6.75)	**0.016**
**Total lean mass female (KG), mean (SD)**	38.46 (5.29)	39.90 (4.65)	37.71 (5.44)	**<0.001**	39.09 (4.56)	38.25 (5.50)	**0.007**
**Total fat mass male (KG), mean (SD)**	23.53 (6.89)	28.64 (5.42)	21.81 (6.47)	**<0.001**	29.44 (4.88)	23.22 (6.84)	**<0.001**
**Total fat mass female (KG), mean (SD)**	29.19 (7.89)	33.35 (6.61)	27.02 (7.63)	**<0.001**	32.66 (7.16)	27.98 (7.78)	**<0.001**
**% fat mass male, mean (SD)**	30.21 (5.46)	33.73 (3.61)	29.03 (5.47)	**<0.001**	36.58 (3.18)	29.87 (5.35)	**<0.001**
**% fat mass female, mean (SD)**	41.47 (5.03)	44.16 (3.38)	40.07 (5.18)	**<0.001**	44.08 (3.70)	40.57 (5.11)	**<0.001**
**Frailty phenotype**							
**Robust, n (%)**	1051 (68.16)	265 (57.11)	786 (72.91)	**<0.001**	123 (48.81)	928 (71.94)	**<0.001**
**Prefrail, n (%)**	440 (28.53)	177 (38.15)	263 (24.40)		109 (43.25)	331 (25.66)	
**Frail, n (%)**	51 (3.31)	22 (4.74)	29 (2.69)		20 (7.94)	31 (2.40)	
**FTS5**							
**Score, mean (SD)**	15.54 (7.14)	19.12 (6.88)	14.00 (6.68)	**<0.001**	23.42 (6.46)	14.04 (6.21)	**<0.001**
**% frail, n (%)**	158 (10.28)	85 (18.32)	73 (6.80)	**<0.001**	93 (37.65)	65 (5.04)	**<0.001**
**Katz Index**							
**Score, mean (SD)**	5.78 (0.58)	5.78 (0.46)	5.79 (0.63)	**0.011**	5.64 (0.65)	5.81 (0.57)	**<0.001**
**% dependency, n (%)**	254 (16.50)	95 (20.47)	159 (14.79)	**0.012**	73 (29.08)	181 (14.05)	**<0.001**
**Mortality, n (%)**	137 (8.79)	42 (8.94)	95 (8.72)	0.8966	26 (10.20)	111 (8.51)	0.4068

**Legend: BMI:** Body Mass Index. **DEXA**: Dual-Energy X-ray Absorptiometry. **EASO:** European Association for the Study of Obesity. **ESPEN:** European Society for Clinical Nutrition and Metabolism. **FTS**: Frailty Trait Scale. **FSOC**: Former Sarcopenic Obesity Criteria. **In bold:** *p*-value < 0.05. **SD**: Standard Deviation.

**Table 3:** Comparison of diagnostic test accuracy metrics according to EASO/ESPEN and FSOC.

Diagnostic Test	EASO/ESPEN vs. FSOC
**Kappa Index (95% CI)**	0.42 (0.36, 0.47)
**Sensitivity (95% CI)**	0.75 (0.70, 0.81)
**Especificity (95% CI)**	0.79 (0.77, 0.81)
**Youden’s J Statistic**	0.55
**+PV (95% CI)**	0,42 (0.37, 0.46)
**−PV (95% CI)**	0.94 (0.92, 0.96)
**+LR**	3.655
**−** **LR**	0.306
**False Negative Rate**	0.242
**False Positive Rate**	0.207
**Diagnostic Odds Ratio**	11.958

**+LR** = Positive Likelihood Ratio; **−LR** = Negative Likelihood Ratio; **+PV** = Positive Predictive Value; **−PV** = Negative Predictive Value.

**Table 4:** Associations between sarcopenic obesity definitions and prevalent frailty and disability.

	M1	M2	M3	M4
**Frailty Phenotype**				
**Variable**	OR (95%CI)	OR (95%CI)	OR (95%CI)	OR (95%CI)
**EASO**	2.02 (1.61, 2.54) **	1.91 (1.51, 2.42) **	1.70 (1.34, 2.17) **	1.70 (1.33, 2.16) **
**FSOC**	2.69 (2.04, 3.54) **	2.51 (1.86, 3.39) **	2.29 (1.68, 3.11) **	2.20 (1.61, 3.00) **
**Ref. EASO-/FSOC-**				
**EASO+/FSOC+**	3.49 (2.55, 4.78) **	3.24 (2.31, 4.55) **	2.89 (2.04, 4.07) **	2.79 (1.97, 3.95) **
**EASO+/FSOC−**	1.38 (1.04, 1.85) *	1.36 (1.01, 1.83) *	1.20 (0.88, 1.62)	1.21 (0.89, 1.64)
**EASO−/FSOC+**	1.49 (0.85, 2.61)	1.36 (0.76, 2.45)	1.19 (0.66, 2.17)	1.12 (0.61, 2.04)
**FTS5 (binary)**				
**Variable**	OR (95%CI)	OR (95%CI)	OR (95%CI)	OR (95%CI)
**EASO**	3.07 (2.20, 4.29) **	2.58 (1.82, 3.66) **	2.35 (1.65, 3.34) **	2.29 (1.60, 3.27) **
**FSOC**	11.38 (7.95, 16.29) **	8.16 (5.55, 11.98) **	7.55 (5.10, 11.17) **	6.82 (4.59, 10.14) **
**Ref. EASO−/FSOC-**				
**EASO+/FSOC+**	11.14 (7.49, 16.57) **	8.03 (5.27, 12.22) **	7.28 (4.75, 11.17) **	6.65 (4.31, 10.25) **
**EASO+/FSOC−**	0.67 (0.34, 1.34)	0.64 (0.32, 1.28)	0.57 (0.28, 1.15)	0.58 (0.28, 1.16)
**EASO−/FSOC+**	8.76 (4.68, 16.42) **	5.77 (2.97, 11.20) **	5.02 (2.53, 9.95) **	4.37 (2.18, 8.73) **
**FTS5 (Continuous)**				
**Variable**	Beta (95%CI)	Beta (95%CI)	Beta (95%CI)	Beta (95%CI)
**EASO**	5.11 (4.38, 5.85) **	4.20 (3.55, 4.86) **	3.69 (3.04, 4.34) **	3.63 (3.00, 4.27) **
**FSOC**	9.38 (8.53, 10.23) **	7.35 (6.53, 8.17) **	6.88 (6.07, 7.68) **	6.57 (5.77, 7.38) **
**Ref. EASO−/FSOC−**				
**EASO+/FSOC+**	10.10 (9.15, 11.06) **	8.12 (7.22, 9.03) **	7.57 (6.69, 8.46) **	7.30 (6.41, 8.18) **
**EASO+/FSOC−**	2.34 (1.51, 3.17) **	2.28 (1.52, 3.03) **	1.79 (1.05, 2.54) **	1.86 (1.12, 2.60) **
**EASO−/FSOC+**	9.07 (7.37, 10.76) **	6.72 (5.15, 8.30) **	6.21 (4.66, 7.75) **	5.82 (4.29, 7.36) **
**Disability**				
	OR (95%CI)	OR (95%CI)	OR (95%CI)	OR (95%CI)
**EASO**	1.48 (1.12, 1.97) **	1.28 (0.96, 1.71)	1.15 (0.86, 1.55)	1.13 (0.84, 1.52)
**FSOC**	2.51 (1.83, 3.44) **	1.82 (1.29, 2.55) **	1.63 (1.15, 2.30) **	1.52 (1.07, 2.16) *
**EASO (−)/FSOC (−)**	Ref.	Ref.	Ref.	Ref.
**EASO (+)/FSOC (+)**	2.59 (1.82, 3.69) **	1.88 (1.29, 2.74) **	1.65 (1.13, 2.41) *	1.55 (1.05, 2.28) *
**EASO (+)/FSOC (−)**	0.96 (0.65, 1.42)	0.94 (0.63, 1.40)	0.85 (0.57, 1.27)	0.86 (0.57, 1.28)
**EASO (−)/FSOC (+)**	2.17 (1.17, 4.01) *	1.50 (0.79, 2.84)	1.31 (0.68, 2.51)	1.21 (0.63, 2.34)

* *p* < 0.05; ** *p* < 0.01. **Legend: M1:** unadjusted model; **M2**: model adjusted by age and sex; **M3**: M2 plus Charlson index and polypharmacy; **M4**: M3 plus educational level. **EASO:** European Association for the Study of Obesity. **ESPEN:** European Society for Clinical Nutrition and Metabolism. **FSOC**: Former Sarcopenic Obesity Criteria.

**Table 5:** Associations between sarcopenic obesity definitions and incident frailty.

	M1	M2	M3	M4
**Frailty Phenotype**				
**Variable**	OR (95%CI)	OR (95%CI)	OR (95%CI)	OR (95%CI)
**EASO**	1.61 (1.13, 2.30) **	1.37 (0.95, 1.98)	1.21 (0.83, 1.75)	1.21 (0.83, 1.77)
**FSOC**	2.61 (1.69, 4.03) **	2.00 (1.25, 3.19) **	1.83 (1.13, 2.95) *	1.70 (1.05, 2.75) *
**Ref. EASO−/FSOC−**				
**EASO+/FSOC+**	2.77 (1.65, 4.64) **	2.03 (1.17, 3.52) *	1.82 (1.03, 3.19) *	1.73 (0.98, 3.04)
**EASO+/FSOC−**	1.31 (0.85, 2.03)	1.22 (0.79, 1.91)	1.05 (0.67, 1.65)	1.07 (0.68, 1.69)
**EASO−/FSOC+**	2.75 (1.32, 5.76) **	2.23 (1.04, 4.81) *	1.93 (0.88, 4.20)	1.73 (0.79, 3.80)
**FTS5 (binary)**				
**Variable**	OR (95%CI)	OR (95%CI)	OR (95%CI)	OR (95%CI)
**EASO**	1.55 (1.00, 2.41)	1.29 (0.82, 2.04)	1.15 (0.72, 1.82)	1.10 (0.68, 1.76)
**FSOC**	5.26 (3.27, 8.47) **	3.67 (2.22, 6.08) **	3.28 (1.96, 5.49) **	2.94 (1.74, 4.94) **
**Ref. EASO−/FSOC−**				
**EASO+/FSOC+**	4.39 (2.50, 7.72) **	3.02 (1.67, 5.46) **	2.65 (1.45, 4.84) **	2.39 (1.30, 4.40) **
**EASO+/FSOC−**	0.95 (0.51, 1.79)	0.87 (0.46, 1.65)	0.76 (0.40, 1.45)	0.74 (0.39, 1.44)
**EASO−/FSOC+**	8.10 (3.70, 17.76) **	5.46 (2.39, 12.45) **	4.62 (1.96, 10.91) **	3.88 (1.65, 9.15) **
**FTS5 (Worsening by 2.5 points)**				
**Variable**	OR (95%CI)	OR (95%CI)	OR (95%CI)	OR (95%CI)
**EASO**	0.93 (0.70, 1.22)	0.96 (0.72, 1.28)	0.91 (0.69, 1.22)	0.95 (0.71, 1.27)
**FSOC**	1.86 (1.28, 2.71) **	1.76 (1.19, 2.59) **	1.76 (1.19, 2.60) **	1.67 (1.12, 2.47) *
**Ref. EASO−/FSOC−**				
**EASO+/FSOC+**	1.51 (0.99, 2.32)	1.48 (0.95, 2.29)	1.45 (0.93, 2.25)	1.43 (0.91, 2.23)
**EASO+/FSOC−**	0.84 (0.61, 1.16)	0.89 (0.63, 1.24)	0.83 (0.59, 1.16)	0.86 (0.61, 1.22)
**EASO−/FSOC+**	2.85 (1.49, 5.47) **	2.61 (1.33, 5.12) **	2.57 (1.29, 5.11) **	2.25 (1.13, 4.47) *

* *p* < 0.05; ** *p* < 0.01. **Legend: M1:** unadjusted model; **M2:** model adjusted by age and sex; **M3**: M2 plus Charlson index and polypharmacy; **M4:** M3 plus educational level. **EASO:** European Association for the Study of Obesity. **ESPEN:** European Society for Clinical Nutrition and Metabolism. **FTS:** Frailty Trait Scale. **FSOC**: Former Sarcopenic Obesity Criteria.

**Table 6:** Associations between sarcopenic obesity definitions and worsening disability and mortality.

Variable	Model 1	Model 2	Model 3	Model 4
**Worsening disability**	OR (95%CI)	OR (95%CI)	OR (95%CI)	OR (95%CI)
**EASO**	1.00 (0.76, 1.32)	0.88 (0.67, 1.17)	0.85 (0.64, 1.13)	0.85 (0.63, 1.13)
**FSOC**	1.43 (1.03, 1.99) *	1.12 (0.79, 1.59)	1.09 (0.77, 1.56)	1.05 (0.73, 1.50)
**EASO (−)/FSOC (−)**	Ref.	Ref.	Ref.	Ref.
**EASO (+)/FSOC (+)**	1.27 (0.87, 1.85)	0.98 (0.66, 1.46)	0.95 (0.64, 1.42)	0.92 (0.62, 1.38)
**EASO (+)/FSOC (−)**	0.90 (0.63, 1.27)	0.86 (0.61, 1.23)	0.82 (0.58, 1.18)	0.83 (0.58, 1.18)
**EASO (−)/FSOC (+)**	1.98 (1.06, 3.69) *	1.53 (0.80, 2.90)	1.45 (0.76, 2.76)	1.36 (0.71, 2.63)
**Mortality**	HR (95%CI)	HR (95%CI)	HR (95%CI)	HR (95%CI)
**EASO**	0.96 (0.67, 1.40)	0.93 (0.64, 1.36)	0.85 (0.58, 1.24)	0.85 (0.58, 1.24)
**FSOC**	1.17 (0.76, 1.81)	1.35 (0.85, 2.14)	1.20 (0.75, 1.91)	1.17 (0.73, 1.87)
**EASO (−)/FSOC (−)**	Ref.	Ref.	Ref.	Ref.
**EASO (+)/FSOC (+)**	1.01 (0.60, 1.70)	1.11 (0.65, 1.90)	0.96 (0.56, 1.66)	0.94 (0.55, 1.62)
**EASO (+)/FSOC (−)**	1.00 (0.63, 1.58)	0.93 (0.59, 1.48)	0.87 (0.55, 1.38)	0.88 (0.56, 1.40)
**EASO (−)/FSOC (+)**	1.80 (0.87, 3.70)	2.55 (1.18, 5.50) *	2.30 (1.06, 5.01) *	2.28 (1.05, 4.96) *

* *p* < 0.05. **Legend: M1:** unadjusted model; **M2:** model adjusted by age and sex; **M3**: M2 plus Charlson index and polypharmacy; **M4:** M3 plus educational level. **EASO:** European Association for the Study of Obesity. **ESPEN:** European Society for Clinical Nutrition and Metabolism. **FSOC**: Former Sarcopenic Obesity Criteria.

## Data Availability

The data presented in this study are available on request from the corresponding author.

## Supplementary Materials

The following supporting information can be downloaded at: https://www.mdpi.com/article/10.3390/nu16193315/s1, Table S1. FTS-5 short-form scoring table. Table S2. Demographic characteristics of study population and classification according to EASO/ESPEN and FSOC. Table S3. Incident adverse events according to the presence or absence of sarcopenic obesity according to the EASO/ESPEN criteria and FSOC. Table S4. Incident adverse events according to different allocation criteria.
