# Supplementary material for: The Performance and Associated Risks of the Criteria for Sarcopenic Obesity Proposed by the European Association for the Study of Obesity in a Geriatric Population
Source: Nutrients. 2024 Sep 30;16(19):3315. doi: 10.3390/nu16193315 (PMC11478913; doi:10.3390/nu16193315)
Supplement: Supplementary file 1 [file nutrients-16-03315-s001.zip › nutrients-3155285-supplementary.pdf]

<u>Supplementary Tables</u>

Supplementary Table S1. FTS-5 Short Form Scoring Table

**Score**	**BMI, kg/m ^{2}**		**PASE**	**Gait Speed, s** *	**Grip Strength, kg**		**Score**	**Progressive Romberg**	
					Women	Men		Position	Seconds
**0**	23.01-26.99		>194	<2.45	>22	>29	0	Tandem	≥10
**1**	27-28.99	21.01-23	174.61-194	2.45-2.99	19.81-22	26.11-29	2.5	Tandem	3.01-9.99
**2**	29-30.99	19.01-21	155.21-174.6	3.00-3.54	17.61-19.8	23.21-26.1	5	Tandem	≤3
**3**	31-32.99	17.01-19	135.81-155.2	3.55-4.09	15.41-17.6	20.31-23.2		Semitandem	≥10
**4**	33-34.99	15.01-17	116.41-135.8	4.10-4.64	13.21-15.4	17.41-20.3	7.5	Semitandem	<10
**5**	35-36.99	13.01-15 †	97.01-116.4	4.65-5.19	11.01-13.2	14.51-17.4		Side by side	≥10
**6**	37-38.99	11.01-13 †	77.61-97	5.20-5.74	8.81-11.0	11.61-14.5	10	Side by side	>10
**7**	39-40.99	NA	58.21-77.6	5.75-6.29	6.61-8.8	8.71-11.6			
**8**	41-42.99	NA	38.81-58.2	6.30-6.84	4.41-6.6	5.81-8.7			
**9**	43-44.99	NA	19.41-38.8	6.85-7.39	2.21-4.4	2.91-5.8			
**10**	≥45	NA	0-19.4	≥7.4	0-2.2	0-2.9			

PASE: Physical Activity Scale for the Elderly.

FTS $_5$ includes all the items of the table (range 0-50), and frail participants are those with FTS $_5$ scores >25.

∗ Gait speed refers to time in accomplish 3-metres at usual pace.

† Model estimation.

Modified of: García-García FJ, Carnicero JA, Losa-Reyna J, Alfaro-Acha A, Castillo-Gallego C, Rosado-Artalejo C, Gutiérrrez-Ávila G, Rodriguez-Mañas L. Frailty Trait Scale-Short Form: A Frailty Instrument for Clinical Practice. J Am Med Dir Assoc. 2020 Sep;21(9):1260-1266.e2. doi: 10.1016/j.jamda.2019.12.008. Epub 2020 Jan 29. PMID: 32005416.

Supplementary Table S2. Demographics characteristics of study population and classification over EASO/ESPEN and FSOC Criteria.

Variable	All	EASO (+) / FSOC (+) Positive Both	EASO (+) / FSOC (-) Positive New	EASO (-) / FSOC (+) Positive Old	EASO (-) / FSOC (-) Negative Both	p-value
**N**	1559	197	273	58	1031	
**Age (years), mean (SD)**	74.79 (5.76)	76.66 (5.00)	74.14 (4.72)	77.52 (5.60)	74.44 (6.04)	**<0.001**
**Gender (male), n (%)**	710 (45.54)	33 (16.75)	146 (53.48)	3 (5.17)	528 (51.21)	**<0.001**
**Charlson Index Score, mean (SD)**	1.19 (1.63)	1.52 (1.94)	1.32 (1.51)	1.21 (1.37)	1.09 (1.60)	**<0.001**
**Number of Drugs, mean (SD)**	4.89 (2.91)	6.34 (2.98)	5.43 (2.64)	6.10 (2.72)	4.40 (2.84)	**<0.001**
**BMI, mean (SD)**	29.18 (4.56)	33.02 (3.97)	31.35 (3.41)	33.16 (5.06)	27.65 (4.08)	**<0.001**
**DEXA parameters**						
**Total lean mass male (Kg) , mean (SD)**	50.74 (6.70)	48.24 (5.09)	54.67 (5.85)	51.30 (6.78)	49.80 (6.60)	**<0.001**
**Total lean mass female (Kg) , mean (SD)**	38.46 (5.29)	39.06 (4.55)	40.97 (4.58)	39.15 (4.65)	37.56 (5.51)	**<0.001**
**Total fat mass male (Kg), mean (SD)**	23.53 (6.89)	29.30 (4.89)	28.49 (5.54)	30.99 (5.45)	21.76 (6.44)	**<0.001**
**Total fat mass female (Kg), mean (SD)**	29.19 (7.89)	33.22 (6.70)	33.53 (6.50)	30.98 (8.22)	26.59 (7.44)	**<0.001**
**% Fat mass male, mean (SD)**	30.21 (5.46)	36.58 (3.24)	33.08 (3.38)	36.56 (2.97)	28.98 (5.46)	**<0.001**
**% Fat mass female, mean (SD)**	41.47 (5.03)	44.58 (3.31)	43.63 (3.40)	42.59 (4.39)	39.80 (5.19)	**<0.001**
**Frailty Phenotype**						
**Robust, n (%)**	1051 (68.16)	86 (44.10)	179 (66.54)	37 (64.91)	749 (73.36)	**<0.001**
**Prefrail, n (%)**	440 (28.53)	92 (47.18)	85 (31.60)	17 (29.82)	246 (24.09)	
**Frail, n (%)**	51 (3.31)	17 (8.72)	5 (1.86)	3 (5.26)	26 (2.55)	
**FTS5**						
**Score, mean (SD)**	15.54 (7.14)	23.65 (6.47)	15.89 (5.14)	22.61 (6.44)	13.54 (6.37)	**<0.001**
**% Frail, n (%)**	158 (10.28)	75 (38.86)	10 (3.69)	18 (33.33)	55 (5.40)	**<0.001**
**Katz Index**						
**Score, mean (SD)**	5.78 (0.58)	5.66 (0.56)	5.86 (0.34)	5.56 (0.89)	5.80 (0.61)	**<0.001**
**% dependency, n (%)**	254 (16.50)	58 (29.90)	37 (13.70)	15 (26.32)	144 (14.15)	**<0.001**

Legend: BMI: Body Mass index. **DEXA**: Dual-Energy X-ray Absorptiometry. **EASO:** European Association for the Study of Obesity. **ESPEN:** European Society for Clinical Nutrition and Metabolism. **FSOC**: Foundation for the National Institutes of Health. **FTS**: Frailty Trait Scale. **In bold:** p-value <0.05. **SD**: Standard Deviation.

Supplementary Table S3. Incident adverse events according to the presence or not of Sarcopenic Obesity according to EASO/ESPEN and FSOC Criteria.

		EASO/ESPEN Criteria			FSOC Criteria		
**Variable**	**All**	**Sarcopenic obesity**	**Non-Sarcopenic Obesity**	**p-value**	**Sarcopenic obesity**	**Non-Sarcopenic Obesity**	**p-value**
**N**	1559	470	1089		255	1304	
**Frailty**							
**Incident frailty (Fried)**	44 (3.55)	13 (3.49)	31 (3.58)	0.938	15 (7.77)	29 (2.77)	**<0.001**
**Incident frailty (FTS5)**	94 (8.12)	34 (10.66)	60 (7.15)	0.061	32 (25.20)	62 (6.01)	**<0.001**
**Worsening 2.5 points (FTS5)**	457 (36.18)	121 (31.68)	336 (38.14)	0.079	74 (37.37)	383 (35.96)	0.762
**Disability**							
**Worsening disability (Katz)**	315 (24.01)	97 (23.83)	218 (24.09)	0.930	22 (10.63)	62 (5.72)	**0.011**
**Mortality**	137 (8.79)	42 (8.94)	95 (8.72)	0.897	26 (10.20)	111 (8.51)	0.407

Legend: EASO: European Association for the Study of Obesity. **ESPEN:** European Society for Clinical Nutrition and Metabolism. **FSOC:** Foundation for the National Institutes of Health. **FTS:** Frailty Trait Scale. **In bold:** p-value <0.05.

Supplementary Table S4. Incident adverse events according to different allocation criteria.

Variable	All	EASO (+) / FSOC (+) Positive Both	EASO (+) / FSOC (-) Positive New	EASO (-) / FSOC (+) Positive Old	EASO (-) / FSOC (-) Negative Both	p-value
**N**	1559	197	273	58	1031	
**Incident Frailty**						
**Incident frailty (Fried)**	44 (3.55)	10 (6.71)	3 (1.34)	5 (11.36)	26 (3.16)	**0.001**
**Incident frailty (FTS5)**	94 (8.12)	21 (22.11)	13 (5.80)	11 (34.38)	49 (6.07)	**<0.001**
**Worsening 2.5 points (FTS5)**	457 (36.18)	52 (33.55)	69 (30.40)	22 (51.16)	314 (37.47)	0.141
**Disability**						
**Worsening disability (Katz)**	315 (24.01)	46 (26.90)	51 (21.61)	17 (36.17)	201 (23.43)	0.243
**Mortality**	137 (8.79)	18 (9.14)	24 (8.79)	8 (13.79)	87 (8.44)	0.610

Legend: EASO: European Association for the Study of Obesity. **ESPEN:** European Society for Clinical Nutrition and Metabolism**. FSOC:** Foundation for the National Institutes of Health. **FTS:** Frailty Trait Scale. **In bold:** p-value <0.05.
